# Expression and Significance of Neuroligins in Myenteric Cells of Cajal in Hirschsprung's Disease

**DOI:** 10.1371/journal.pone.0067205

**Published:** 2013-06-28

**Authors:** Jian Wang, Yaru Mou, Qiangye Zhang, Fan Zhang, Hongchao Yang, Wentong Zhang, Aiwu Li

**Affiliations:** 1 Department of Pediatric Surgery, Qilu Hospital, Shandong University, Jinan, Shandong, China; 2 Department of Cardiology, Provincial Hospital Affiliated to Shandong University, Jinan, Shandong, China; 3 Department of E.N.T, Qilu Hospital, Shandong University, Jinan, Shandong, China; The Chinese University of Hong Kong, Hong Kong

## Abstract

**Purpose:**

The aim of this study was to investigate the expression and significance of neuroligins in myenteric cells of Cajal (ICC-MY) in Hirschsprung’s disease (HSCR).

**Methods:**

Longitudinal muscle with adherent myenteric plexus (LMMP) from surgical excision waste colon of HSCR children were prepared by peeling off the mucous layer, sub-mucosal layer and circular muscle. Neuroligins, c-Kit (c-Kit-immunoreactivity representing ICC) and their relationship were assessed by double labeling immunofluorescence staining. ICC-MY were dissociated and cultured from LMMP by enzymolysis method, and were purified and analyzed using a combination of magnetic-activated cell sorting (MACS) and flow cytometry (FCM). Western-blot analysis was applied to compare and evaluate the expression levels of neuroligins in ICC-MY which were dissociated from different segments of HSCR (ganglionic colonic segment, transitional colonic segment and aganglionic colonic segment).

**Results:**

Neuroligins and c-Kit were expressed on the same cells (ICC-MY); ICC-MY were dissociated, cultured and purified. For HSCR, neuroligins were expressed significantly in ICC-MY from ganglionic colonic segments, moderately in those from transitional colonic segments and down-regulated significantly in those from aganglionic colonic segments.

**Conclusions:**

Neuroligins were expressed in ICC-MY of human beings, and the expression varies from different segments of HSCR. This abnormal expression might play an important role in the pathogenesis of this disease through affecting the synaptic function of ICC-MY.

## Introduction

Hirschsprung’s disease (HSCR) is a congenital condition that affects 1 of 5,000 human births and is characterized by colonic stasis due to the absence of enteric neurons in the distal gut [1], leading to tonic contraction of the affected segment, intestinal obstruction and massive distension of the proximal bowel (magacolon). In the majority of cases (80%), the aganglionic tract involves the rectum and the sigmoid colon only (short segment HSCR) while in 20% of cases it extends towards the proximal end of the colon (long segment HSCR) [2].

It has been identified that several genes such as RET, SOX10 and EDNRB are involved in the pathogenesis of HSCR in human beings [3]. However, the alteration and abnormal expression of these genes only account for 30% of the cases of HSCR [4].

As important players in the symphony of gut motility, interstitial cells of Cajal (ICC) have a very significant physiological role in orchestrating the normal peristaltic activity of the digestive system and they are the pacemaker cells in gastrointestinal muscles [5], especially the myenteric cells of Cajal (ICC-MY) between the circular and longitudinal muscle layers. ICC-MY are the pacemaker cells trigging the generation of slow waves in the tunica muscularis [6]. ICC can be identified immunohistochemically by the expression of stem cell factor (SCF) receptor (c-Kit, CD117), a tyrosine kinase [7]. Therefore specifically designed c-Kit(CD117) antibodies have been developed to be the marker of ICC and ICC can be not only identified by their ability to bind to c-Kit(CD117) antibodies but also by their clear morphological features[8]–[9]. Also ICC could be purified and analyzed by using a combination of magnetic-activated cell sorting (MACS) and flow cytometry (FCM) by the marker fraction as Kit^+^CD44^+^ [10]–[12].

Although many studies reported that decreased numbers or disrupted networks of ICC were associated with HSCR [13], a further research of the relationship between ICC and HSCR was still needed.

In the central nervous system (CNS), it has been identified that neuroligins which belong to a highly conserved family of cell adhesion molecules are implicated in synapse formation and function [14]. Synapse is the foundation of information transmission among neurons by transporting neurotransmitter. Recent findings on synaptogenesis suggested that neuroligins play an important role in the formation of synapses and their maturation, by forming heterophillic trans-synaptic cell adhesion complex interacting with their presynaptic partners [15].

It is generally accepted that there is bi-directional communication between the CNS and the enteric nervous system (ENS) [16]. Indeed, the brain is continuously informed by afferent nerves detecting gut activity, whereas it is well established that psychological state or stress has a major influence on gut function [16]. Hence, some questions were raised naturally: 1. Were neuroligins expressed on ICC-MY of ENS? 2. Was the expression level of neuroligins abnormal? 3. Whether the abnormal expression of neuroligins was involved in pathogenesis of HSCR?

In order to explore these questions, we investigated the expression of neuroligins in ICC-MY of children patients with HSCR by immunofluorescence and evaluated the expression levels of neuroligins in ICC-MY dissociated from different segments in HSCR by Western-blot analysis.

We hope that our work would be helpful for the research of pathogenesis of HSCR.

## Materials and Methods

### Patients

All protocols of this study were approved by the ethics committee of Qilu Hospital, Shandong University. Informed verbal consent was obtained from the next of kin, caretakers, or guardians on behalf of the minors/children participants involved in our study during preoperative conversation, and related content and consent process were documented in the operation agreement. The experimental samples were collected from the surgical excision waste tissue and no harm had been done to patients at all, so the special written consent was not necessary according to the opinion of the ethics committee of Qilu Hospital, Shandong University.

Fifty patients (2 months-5 years, 35 boys, 15 girls) involved in this study were treated in Department of Pediatric Surgery, Qilu Hospital, Shandong University from 2009 to 2011, who were pathologically confirmed HSCR. The cases of short-segment type and long-segment type were 39 and 11 respectively. All patients were treated with Soave’s pull-through procedure.

### Reagents

The detailed information of primary and secondary antibodies is listed in table 1 and table 2 and other reagents were also available commercially: Polymer Helper (ZSGB-BIO, China); 3,3-diaminobenzidine (DAB) (ZSGB-BIO, China); TRIS (Merck-Belgolabo, Overijse, Belgium); Normal goat serum (Hormonologie Laboratoire, Marloie, Belgium); CollagenaseII (Solarbio, China); Newborn calf serum(Solarbio, China); Trypsin inhibitor (sigma, USA); M119 (Gibco, USA); SCF (Perotech, Canada); CD117 MicroBead Kit human (Miltenyi Biotec, GER); CD44 MicroBead Kit human (Miltenyi Biotec, GER).

**Table 1:** Primary antibody.

Antigen	Antibody	Dilution	Applications	Source
Neuroligin-1,2,3,4	Rabbit-anti-human	1∶500	Detect Nlgns with	Gershon’s lab of
	polyclonal		immunofluorescence	Columbia university
Neuroligin-1,2,3,4	Rabbit-anti-human	1∶200	Detect Nlgns with	Gershon’s lab of
	polyclonal		Western-blot	Columbia university
c-Kit(CD117)	Goat-anti-human	1∶50	Detect Nlgns with	Dako, Glostrup,
	polyclonal		Immunofluorescence/	Denmark
			immunohistochemistry	
c-Kit(CD117)	Goat-anti-human	1∶30	Quantitative analysis	Dako, Glostrup,
	polyclonal		of ICC-MY by FCM	Denmark
CD44	Goat-anti-human	1∶50	Quantitative analysis	Dako, Glostrup,
	polyclonal		of ICC-MY by FCM	Denmark

**Table 2:** Secondary antibody.

Antibody	Dilution	Application	Source
Goat anti-rabbit Texas Red	1∶200	Label Nlgn-1,2,3,4 (Nlgns)	Jackson Immuno-research
			Laboratories
Donkey anti-goat FITC	1∶400	Label c-Kit	Jackson Immuno-research
			Laboratories
Horseradish peroxidase-conjugated goat	1∶1000	Detect Nlgn-1,2,3,4 (Nlgns)	Santa Cruz
anti-rabbit IgG		with Western-blot	
Polyperoxidase-anti-goat IgG	1∶500	Label c-Kit with	ZSGB-BIO
		immunohistochemistry	

### Methods

#### Expression of neuroligins in ICC-MY

Preparation of LMMP: Aganglionic, transitional and ganglionic segments were harvested from the surgical excision waste colon of children patients with HSCR at the length of 3 cm respectively. Specimens were opened along mesentery, and their contents were washed away with ice-cold Krebs-Ringer buffer solution. The mucosa, submucosa and circular muscle were removed by peeling in the dish coated with Sylgard elastomer (Dow Corning Co., Midland, MI) under a stereomicroscope. Each prepared LMMP was divided into three small pieces of sample (each 1 cm×1 cm). One piece which would be used for the isolation and culture of ICC-MY should be freshly prepared, and the other two which would be used for immunohistochemical staining and double immunofluorescence labeling could be stored in PBS containing 0.1% sodium azide at 4°C and would be used within a week.

Identification of ICC-MY on LMMP: Three small samples of LMMP (each 1 cm×1 cm, respectively from aganglionic, transitional and ganglionic segments) were used for the identification of ICC-MY on LMMP by immunohistochemical staining. After being performed in 3% hydrogen peroxide solution (H_2_O_2_), hatching for 10 min to inactivate endogenous peroxidase, the samples were blocked with 3% goat normal serum diluted in 3% Triton-PB for 1 h at room temperature (RT). Anti-c-Kit-receptor (CD117) antibodies were employed as primary antibodies and the slices were incubated in primary antibodies for 24 h at 4°C. PBS served as negative control by omitting the primary antibody. Then Polymer Helper and polyperoxidase-anti-goat IgG were added dropwise in turn and incubated for 20 min and 30 min respectively at 37°C. At last, DAB was added as a chromogen and stained.

Identification of expression of neuroligins in ICC-MY: After the identification of ICC-MY on LMMP, double immunofluorescence labeling technique was applied to identify the expression of neuroligins in ICC-MY on LMMP. Three small patches of LMMP (each 1 cm×1 cm, respectively from aganglionic, transitional and ganglionic segments) were brought to RT rinsed in 10 mM TRIS and 0.15 M sodium chloride, pH 7.4 (TRIS-buffered saline, TBS), containing 0.1% (v/v) Triton X-100 (TBS-TX), and incubated for 1 h at RT in 3% normal goat serum and TBS-TX to reduce background staining. The samples were left overnight at RT in a humid chamber with the primary antibodies (neuroligin1,2,3,4 and c-Kit(CD117)) diluted in TBS-TX, rinsed in TBS and then incubated in the dark for 2 h at RT in TBS containing the donkey anti-goat Texas Red and donkey anti-goat FITC secondary antibodies. After five rinses in TBS, the sections were mounted with UltraCruzTM mounting medium. Selective detection of the green (FITC) and red (Texas red) fluorochromes was detected by laser scanning confocal microscope. For rendering the strong autofluorescence, the red and green dataset were digitally combined. At last, pictures were exported on a Power Mac (Apple, Cupertino, CA) running Freehand (Macromedia, San Francisco, CA) and Illustrator (Adobe, San Francisco, CA) software.

#### Changes of expression level s of neuroligins in ICC-MY

Dissociation and culture of ICC-MY: Three fresh patches of LMMP (each 1 cm×1 cm, respectively from aganglionic, transitional and ganglionic segments) were immersed in 0.01 mol/L PBS which included Penicillin 10^5^ U/L and Streptomycin 100 mg/L for 1 h. After five rinses in PBS, they were immersed into enzymolysis liquid containing CollagenaseII1.3g/L, ATP 0.25 g/L, 10% newborn calf serum and trypsin inhibitor 2 g/L for digestion at 37°C for 1 h. After removing the supernatant, M119 was added and centrifuged at speed of 1000 r/min for 5 min and then removed the supernatant, added M119 and centrifuged at speed of 1000 r/min for 5 min again. At last, M119 was added and after piping and drumming for 10 min, 200 meshes of screen mesh was passed through. After M199 culture medium was added, which included 10% newborn calf serum, SCF 5 µg/L, Penicillin 10^5 ^U/L, Streptomycin 100 mg/L and M199, cells were inoculated onto 6-well plates and cultured at the condition of 37°C and 5% CO_2_ for 24 h. M199 culture medium excluded Penicillin and Streptomycin was used to take place of the original culture medium after 24 h and needed to be replaced every 3–4 days.

Isolation and purification of ICC-MY: In order to isolate and purify ICC-MY, 2-step MACS was used as reported in literature[17]–[18], and the operation was taken according to the directions of MACS kit: CD117 MicroBead human Kit and CD44 MicroBead human Kit. Then most interference cells could be excluded by 2-step sorting, such as neurons and glial cells[17]–[18].

Quantitative analysis of ICC-MY by FCM: Three small samples of cells were drawn from the cells which we had isolated and purified previously (respectively from aganglionic, transitional and ganglionic segments) for quantitative analysis by FCM. Primary antibodies (c-Kit(CD117), CD44) were added and incubate for 20 min at 37. After 2×5 min washes in 0.1 mol/L PBS, donkey anti-goat FITC secondary antibodies were added and incubate for 30 min at 37°C in darkness. After 2×5 min washes in 0.1 mol/L PBS, FCM was used for quantitative analysis.

Identification of ICC-MY: To identify the successful culture of ICC-MY, a small amount of cells was drawn from the cells which we had isolated and purified previously and immunofluorescence labeling technique was used. After supernatant was abandoned, 0.01 mol/L PBS was added to wash the cells 3×5 min. After acetone fixation for 10 min and 3×5 min washes in 0.01 mol/L PBS, the cells were blocked with 10% newborn calf serum at 37°C for 40 min. After 3×5 min washes in 0.01 mol/L PBS, cells were left overnight at 4°C in a humid chamber with the primary antibodies (c-Kit(CD117)), rinsed in 0.01 mol/L PBS and then incubated in the dark for 2 h at 37°C in TBS containing the donkey anti-goat FITC secondary antibodies. Laser confocal microscope was used for observation.

Expression levels of neuroligins in ICC-MY: After isolation and purification of ICC-MY, most cells (except the cells used for FCM analysis and identification of ICC-MY) from the cells which we had isolated and purified previously were used to evaluate the expressing quantity of neuroligins by Western-blot analysis. Protein levels were normalized to c-Kit (ICC-specific protein) but not β-actin commonly used, because we wanted to prove that if neuroligins’ expression was downed-regulated in aganglionic segment, it was not due to a decrease in ICC-MY numbers in this segment. ICC-MY from three different segments of HSCR were lysed respectively with 1×SDS sample buffer (1% Triton X-100; 0.1% SDS; 150 mM Tris-HCL, PH7.5; 1 mM EDTA, PH7.5; 1% sodium deoxycholate; 1% Cocktail; 1 mM Na_3_VO_4_; 0.5 Mm PMSF). 30 µg proteins separated from three different segments were electrophoresed respectively and simultaneously on 10% SDS-PAGE and transferred to PVDF membrane. After blocking with 5% (w/v) nonfat milk and washing with Tris-buffered saline-Tween solution (TBST), membranes were incubated overnight at 4°C with neuroligins (neuroligin1,2,3,4) and c-Kit (CD117) antibodies, respectively. After washing, the blots were incubated with horseradish peroxidase-conjugated goat anti-rabbit IgG for 1 h at RT. After washing with TBST, signals were detected using ECL plus chemiluminescence kit on X-ray film (Millipore Corporation,Billerica, USA). Then the gray level was measured by the image analyzer (ImageJ Launcher Software). Statistical comparison was made with unpaired T tests, with P value less than 0.05 considered significant.

## Results

### Identification of ICC-MY in LMMP

**Figure 1:**
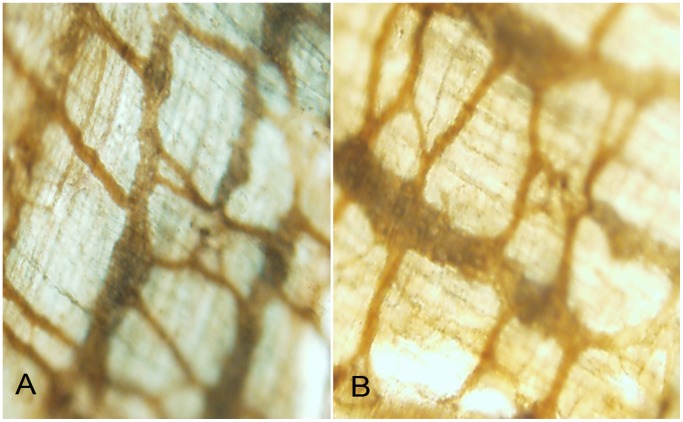
Immunohistochemical staining was performed on LMMP, light microscopy was used for observation. ICC-MY were identified by immunoreactivity for the tyrosine kinase receptor c-Kit(CD117) (brownish yellow, network structure) on LMMP. Figure 1A represented ganglionic colonic segment and Figure 1B represented aganglionic colonic segment. No obvious disruptions of ICC-MY network were showed in aganglionic segments. Magnification 40×.

After immunohistochemical staining was performed on LMMP, light microscopy (magnification, ×40) was used for observation. Figure 1 represented that ICC-MY (c-Kit expressed) were located on LMMP and interwove network structure. Figure 1A represented ganglionic segments and Figure 1B represented aganglionic segments respectively. No obvious disruptions of ICC-MY network were showed in aganglionic segments, which was consistent with the reported literature [19].

### Identification of Expressions of Neuroligins in ICC-MY

**Figure 2:**
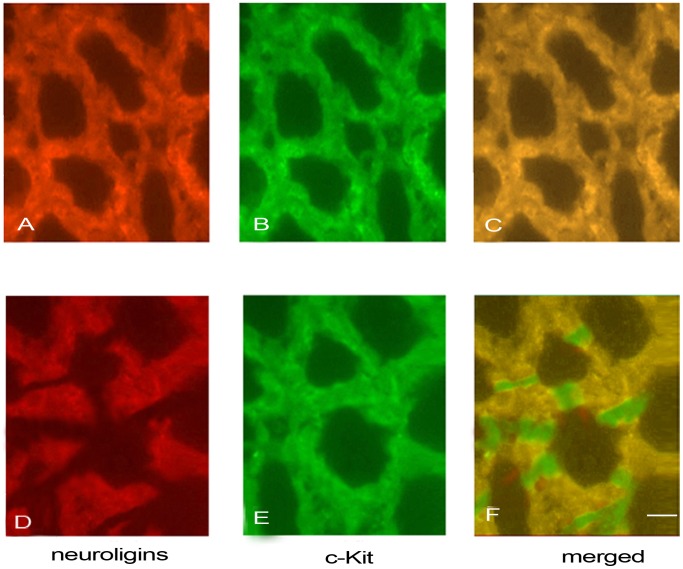
Double-labeled immunofluorescent staining of neuroligins and c-Kit was performed on LMMP. Laser confocal microscope was used for observation. Figure 2A, 2B and 2C represented that in ganglionic segments, neuroligins (Figure 2A, red) were expressed in the same cells (merged, Figure 2C, yellow) in which c-Kit was expressed (Figure 2B, green). Figure 2D, 2E and 2F represented that in aganglionic segments, neuroligins (Figure 2D, red) were expressed in the same cells (merged, Figure 2F, yellow) in which c-Kit was expressed (Figure 2E, green). In aganglionic segments, the expression of neuroligins was down-regulated and neuroligins network was disrupted (Figure 2D), but no obvious disruptions of ICC-MY network were showed (Figure 2E). Scale bars:50 µm.

Double-labeled immunofluorescent staining of neuroligins and c-Kit was performed to identify whether neuroligins were expressed in ICC-MY and laser confocal microscope was used for observation. Figure 2A, 2B and 2C represented that in ganglionic segment, neuroligins (Figure 2A, red) were expressed in the same cells (merged, Figure 2C, yellow) in which c-Kit was expressed (Figure 2B, green), illustrating that neuroligins were expressed in ICC-MY. Figure 2D, 2E and 2F represented that in aganglionic segment, neuroligins (Figure 2D, red) were expressed in the same cells (merged, Figure 2F, yellow) in which c-Kit was expressed (Figure 2E, green). However, in aganglionic segment, the neuroligins were not expressed in all ICC-MY (Figure 2D), and no obvious disruptions of ICC-MY network were showed in this segment (Figure 2E). The results illustrated that neuroligins were both expressed in ICC-MY of aganglionic segments and ganglionic segments. The expression of neuroligins was down-regulated and neuroligins network was disrupted in aganglionic segments of HSCR.

### Quantitative Analysis of ICC-MY by FCM

**Figure 3:**
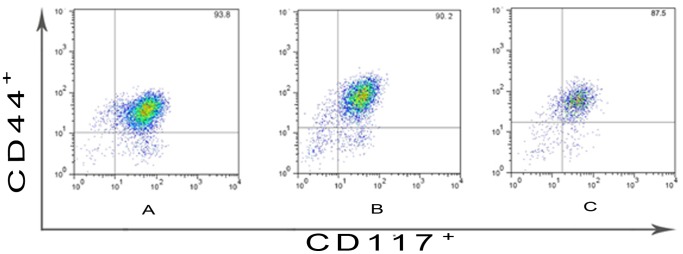
ICC-MY percentages of ganglionic, transitional and aganglionic segments from one case of HSCR were shown by Figure 3A, 3B and 3C, respectively.

**Figure 4:**
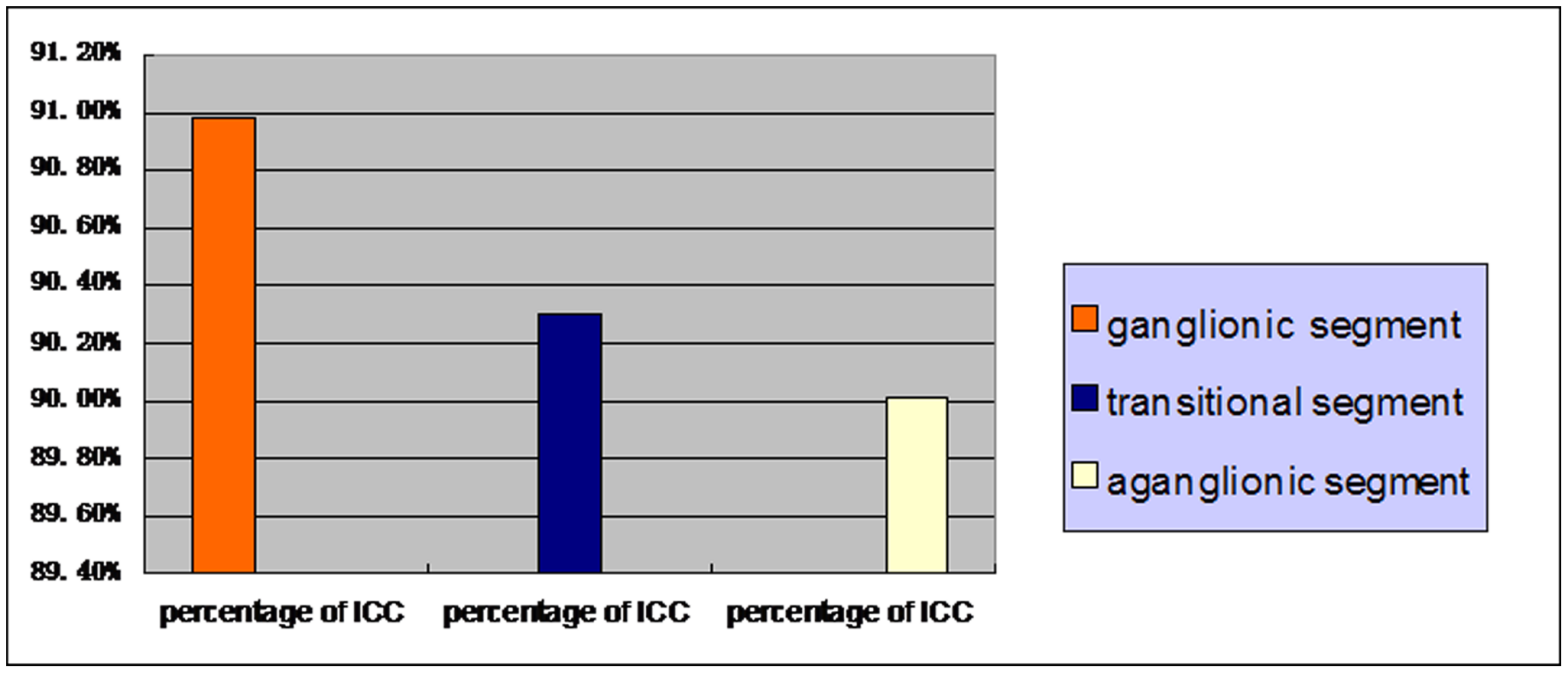
ICC-MY percentages of ganglionic, transitional and aganglionic segments from all 50 cases HSCR and the numerical data are presented as the mean±standard deviation (90.98±3.24, 90.30±3.09, 90.01±3.11, n = 50). Statistical analysis was performed using T test, and P<0.05 was considered statistically significant.

Purity of ICC-MY was analysed by FCM after MACS. ICC-MY percentages of ganglionic, transitional and aganglionic segments from one case of HSCR were showed by Figure 3A, 3B and 3C respectively. Figure 4 represented ICC-MY percentages of ganglionic, transitional and aganglionic segments from all 50 HSCR cases and the numerical data were presented as the mean±standard deviation (90.98±3.24%, 90.30±3.09%, 90.01±3.11%, n = 50). Statistical analysis was performed using T test, and P<0.05 was considered statistically significant. The results illustrated that after MACS, ICC-MY with high purity could be obtained and the result of Western-blot was reliable. And there was no statistical significance among aganglionic, transitional and ganglionic segments (90.01±3.11% vs 90.30±3.09%, P>0.05; 90.01±3.11% vs 90.98±3.24%, P>0.05; 90.30±3.09% vs 90.98±3.24%, P>0.05).

### Identification of ICC-MY

**Figure 5:**
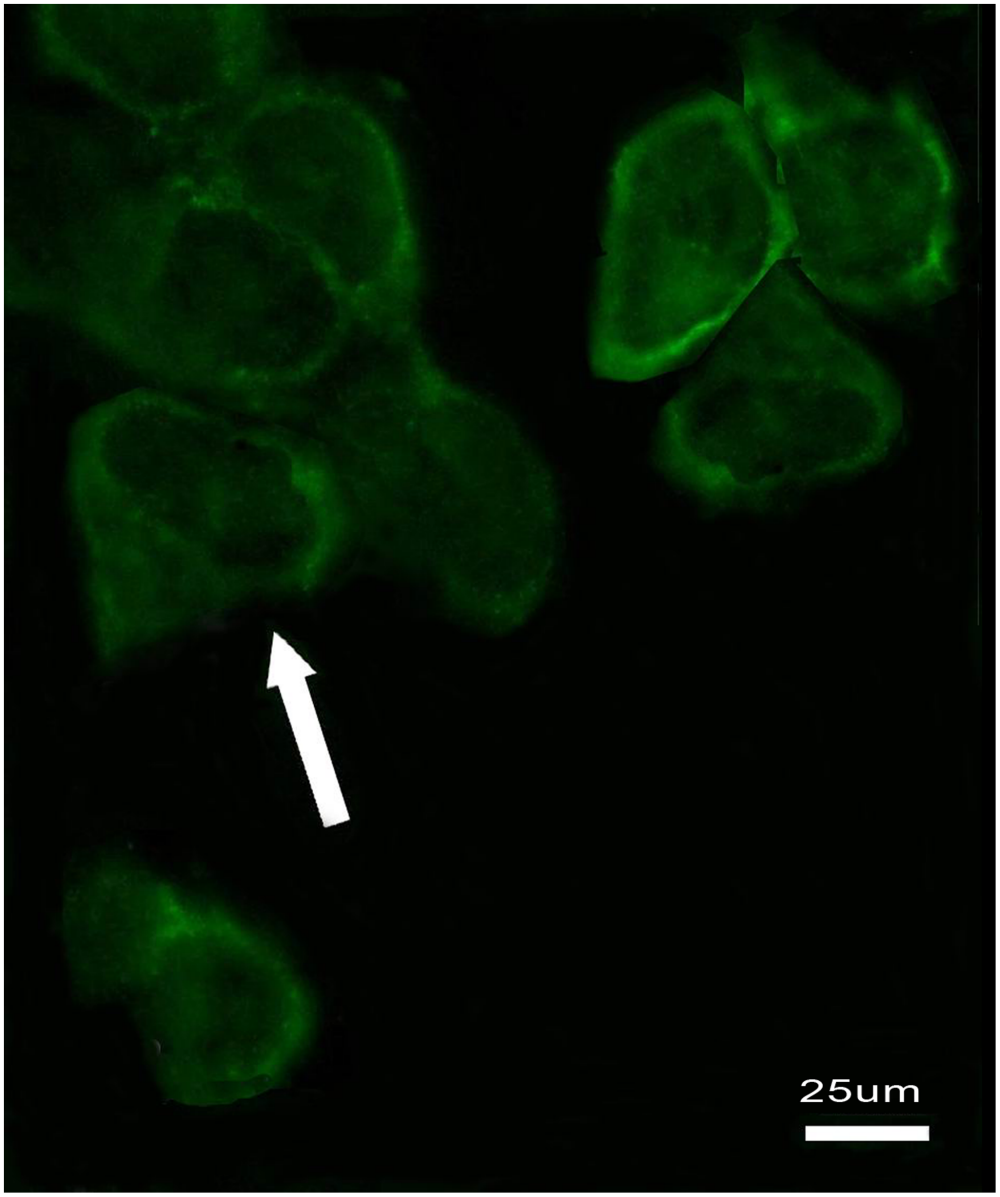
Immunofluorescence labeling was used to identify ICC-MY. c-Kit was expressed in ICC-MY, whose cell bodies and processes were obviously expressed. These cells interact and interwove with each other, whose shape was fusiform or triangular. Scale bars:25 µm.

Immunofluorescence labeling technique was used to identify ICC-MY. Figure 5 represented that c-Kit was expressed in ICC-MY, whose cell bodies and processes were obviously expressed but not cell nuclear. These cells interact and interweaved with each other, whose shape was fusiform or triangular (Figure 5).

### Expression of Neuroligins in ICC-MY from Different Segments

**Figure 6:**
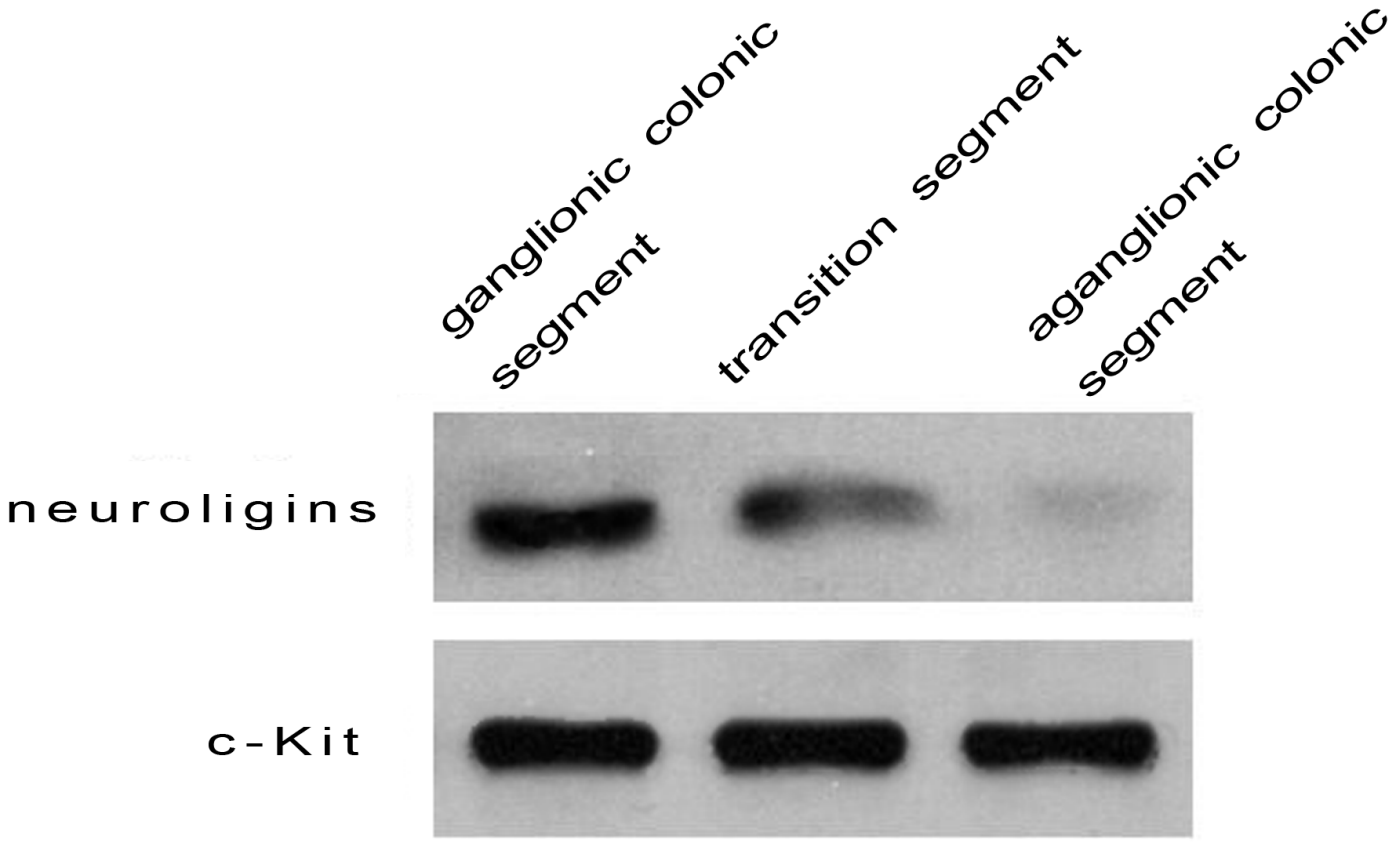
Western blot analysis was used to compare and evaluate the expressing quantity of neuroligins in ICC-MY of different segments in HSCR. Neuroligins were expressed significantly in ICC-MY of ganglionic colonic segment, moderately in transitional segment, and obviously downed-regulated in aganglionic colonic segment.

**Figure 7:**
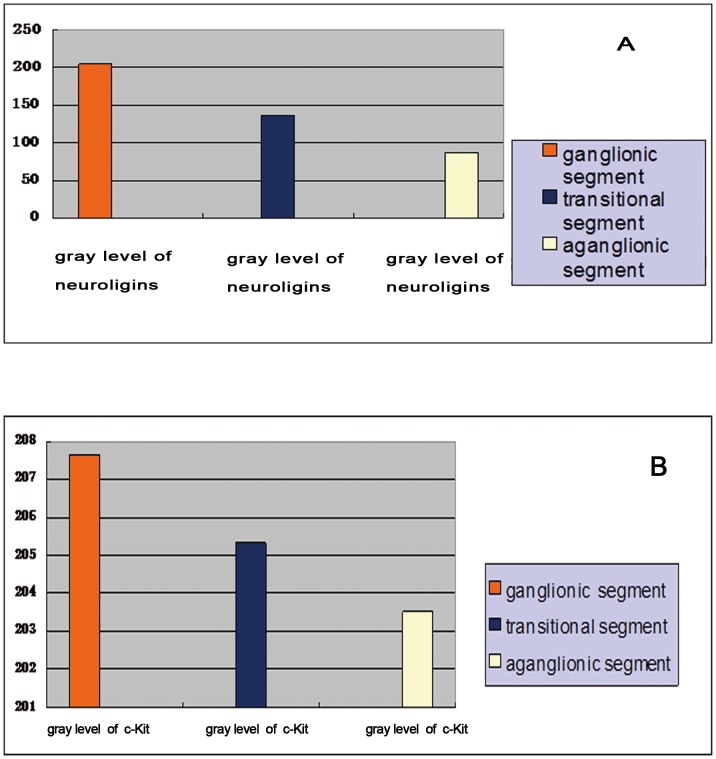
The gray level of neuroligins expressed in Western blot analysis was 204.07±8.81 in ICC-MY of ganglionic colonic segments, 136.13±13.2 in transitional segments and 86.65±4.54 in aganglionic colonic segments, and the difference of gray level had statistical significance (204.07±8.81 vs 136.13±13.2, P<0.05; 136.13±13.2 vs 86.65, P<0.05; 204.07±8.81 vs 86.65±4.54, P<0.05). **(**Figure 7A****).**** The gray level of c-Kit expressed in Western blot analysis was 207.66±13.5 in ICC-MY of ganglionic colonic segments, 205.32±10.75 in transitional segments and 203.52±11.99 in aganglionic colonic segments and there was no statistical difference among aganglionic, transitional and ganglionic colonic segments (207.66±13.5 vs 205.32±10.75, P>0.05; 205.2±10.75 vs 203.52±11.99, P>0.05; 207.66±13.5 vs 203.52±11.99, P>0.05 ). (Figure 7B).

Neuroligins are implicated in synapse formation and function which is the foundation of information transmission among neurons by transporting neurotransmitter. Since ICC-MY expressed neuroligins and ICC-MY with high purity could be obtained based on our experiments above, we wished to observe the expression of neuroligins in ICC-MY from different segments in HSCR by the method of Western blot. Figure 6 and Figure 7 represented that neuroligins were expressed significantly in ICC-MY of ganglionic colonic segments (gray level was 204.07±8.81, n = 50), moderately in transitional colonic segments (gray level was 136.13±13.2, n = 50) and obviously downed-regulated in aganglionic colonic segments (gray level was 86.65±4.54, n = 50), and the difference of gray level had statistical significance (204.07±8.81 vs 136.13±13.2, P<0.05; 136.13±13.2 vs 86.65, P<0.05; 204.07±8.81 vs 86.65, P<0.05). The expression of c-Kit(CD117) had no statistical significance among aganglionic, transitional and ganglionic segments (207.66±13.5 vs 205.32±10.75, P>0.05; 205.2±10.75 vs 203.52±11.99, P>0.05; 207.66±13.5 vs 203.52±11.99, P>0.05 ) (Figure 6, 7). Statistical analysis was performed using T test, and P<0.05 was considered statistically significant. The results represented that neuroligins’ expression was specifically down-regulated in ICC-MY of aganglionic segments, not due to a decrease in ICC numbers in these segments.

### Conclusions

The results above show that neuroligins are expressed in ICC-MY of human beings’ ENS, and for HSCR children, the different expression of neuroligins in ICC-MY from different colon segments may play important role in the pathogenesis of this disease: the obviously downed-regulated expression of neuroligins in ICC-MY of aganglionic colonic segment in HSCR can cause the abnormal synaptic function of ICC-MY, and may further result in the weakness or even disappearance of colorectal peristalsis, intestinal obstruction and massive distension of the proximal bowel (magacolon).

## Discussion

Normal gastrointestinal motility results from the coordinated interplay of multiple cooperating mechanisms, both intrinsic and extrinsic to the gastrointestinal tract [20]. A fundamental component of this activity is an omnipresent electrical activity termed slow waves, which is generated and propagated by the ICC [20]. Several different classes of ICC are now described, based on their anatomical site [21]. A specific receptor tyrosine kinase (c-Kit) is a well-established marker for ICC [22].

The ICC-MY surrounding the myenteric plexus are the primary pacemakers of the gut [20]. Abnormal distribution of ICC has been reported in several diseases and abnormal function of ICC might actually be involved in many disorders of gastrointestinal transit [23], including infantile hypertrophicpyloric stenosis [23] and HSCR[24]–[25]. Although cumulative evidence suggests that HSCR is the consequence of multiple gene interactions that modulate the ability of enteric neural crest cells to populate the developing gut [26], the pathophysiological basis of colonic aperistalsis in HSCR is still not fully understood and a further research of the relationship between ICC and HSCR is still needed.

In the CNS, coculture experiments using nonneuronal cell lines expressing members of the neuroligins gene families revealed that neuroligins specifically induce presynaptic differentiation and also help to drive the functional of synapses [27], which is the foundation of information transmission among neurons by transporting neurotransmitter. A number of studies have shown that the vertebrate neuroligin-neurexin complex appears to influence synapse specificity through excitatory versus inhibitory synapse development, and thus is predicted to influence the excitatory/inhibitory synapse ratio in the brain[28]–[31]. The combinatorial nature of neurexin/neuroligin interactions is believed to be key to neuronal plasticity mechanisms such as learning and memory, and also a likely mediator of mental disorders such as autism [32]. A mismatch of neurexin and neuroligin partners across synapses in the brain presumably leads to loss of synaptic plasticity and/or erroneous wiring, resulting in behavioural and cognitive deficiencies [33].

Recent evidence shows that the bi-directional communication along the brain-gut axis is not only confined to gut digestion and motility, but also involves immunological mechanisms, i.e. the immune system affects neuromuscular function whereas the nervous system has a major modulatory input on the immune system [16].

On the basis of this bi-directional communication between the CNS and the ENS and present study about neuroligin-neurexin, our prophase research reveals that [34] neuroligins are expressed on the postsynaptic neurons in mesenteric plexus of mouse, guinea pigs and human beings with HSCR. Then on the basis on our prophase research, we go on our study and our findings demonstrate that in this study: 1. neuroligins are expressed outside the central nervous system; 2. neuroligins are expressed in ICC-MY dissociated and cultured from the diseased colon of HSCR children; 3. ICC-MY from aganglionic colonic segments express the least neuroligins, the ganglionic colonic segment express the most, and the expression of transitional segments is in the middle.

According to our present research results, it can be inferred that the abnormality of neuroligins is closely related to HSCR, and we could conclude that the alteration and abnormal expression of neuroligins may play important role in the pathogenesis of HSCR through affecting the synaptic function of ICC-MY.

We believe that in the future, it will be important to identify the specific roles in cell functional maturation and function of each subtype of neuroligins which was expressed in ICC-MY or in all the ICC. This may lead to new methods and new therapies for the treatment of HSCR and we hope that our work would be helpful for that.
